# Wearable, Integrated EEG–fNIRS Technologies: A Review

**DOI:** 10.3390/s21186106

**Published:** 2021-09-12

**Authors:** Julie Uchitel, Ernesto E. Vidal-Rosas, Robert J. Cooper, Hubin Zhao

**Affiliations:** 1 DOT-HUB, Department of Medical Physics and Biomedical Engineering, University College London, London WC1E 6BT, UK; ju236@cam.ac.uk (J.U.); ernesto.vidal@ucl.ac.uk (E.E.V.-R.); robert.cooper@ucl.ac.uk (R.J.C.); 2 Department of Paediatrics, University of Cambridge, Cambridge CB2 0QQ, UK; 3 James Watt School of Engineering, University of Glasgow, Glasgow G12 8QQ, UK

**Keywords:** fNIRS, EEG, diffuse optical tomography, wearable, multimodal, integrated

## Abstract

There has been considerable interest in applying electroencephalography (EEG) and functional near-infrared spectroscopy (fNIRS) simultaneously for multimodal assessment of brain function. EEG–fNIRS can provide a comprehensive picture of brain electrical and hemodynamic function and has been applied across various fields of brain science. The development of wearable, mechanically and electrically integrated EEG–fNIRS technology is a critical next step in the evolution of this field. A suitable system design could significantly increase the data/image quality, the wearability, patient/subject comfort, and capability for long-term monitoring. Here, we present a concise, yet comprehensive, review of the progress that has been made toward achieving a wearable, integrated EEG–fNIRS system. Significant marks of progress include the development of both discrete component-based and microchip-based EEG–fNIRS technologies; modular systems; miniaturized, lightweight form factors; wireless capabilities; and shared analogue-to-digital converter (ADC) architecture between fNIRS and EEG data acquisitions. In describing the attributes, advantages, and disadvantages of current technologies, this review aims to provide a roadmap toward the next generation of wearable, integrated EEG–fNIRS systems.

## 1. Introduction

Multimodal functional neuroimaging allows for the concurrent assessment of two or more complementary features of brain activity. Because they represent two entirely different physiological processes, many multimodal functional neuroimaging studies combine a method that measures the neuronal electrical activity of the brain with a method that measures hemodynamic activity, to provide a more comprehensive picture of brain function.

In multimodal studies, the most common method to assess the brain’s electrical activity is electroencephalography (EEG). EEG passively records electrical signals associated with cortical neuronal activity using scalp electrodes. It is routinely used in studies of brain function due to its noninvasiveness, cost-effectiveness, portability, and capacity for long-term monitoring. Common methods to assess the brain’s hemodynamic activity include position emission tomography (PET), functional magnetic resonance imaging (fMRI), and functional near-infrared spectroscopy (fNIRS) [1,2,3,4,5]. Although fMRI has been successfully applied in multimodal studies (EEG–fMRI [3]), the limitations of this technologies (such as its fixed location, high cost, and patient discomfort) inhibit its application to certain patient and participant groups (e.g., neonates, young children, and the elderly) and prevents use outside of a hospital or laboratory environment. This is not the case for fNIRS, which is a low-cost, portable, and noninvasive technique that assesses changes in cortical hemodynamic activity in the brain using near-infrared (NIR) light [6]. 

For these reasons, multimodal studies of brain function have increasingly used EEG and fNIRS simultaneously. EEG and fNIRS are both low cost, portable, and potentially appropriate for long-term subject monitoring in clinical and nonclinical settings. Together, these technologies therefore allow for concurrent assessment of electrical and hemodynamic activity in the brain. Multimodal EEG and fNIRS systems, or EEG–fNIRS systems, have been demonstrated in diverse applications in neuroscience and clinical neurological care [7,8,9]. Studies of brain–computer interfaces (BCI) frequently employ combined EEG–fNIRS systems to increase the amount of available information from the brain and improve classification accuracy [5,10,11,12,13,14]. Clinically, EEG–fNIRS has demonstrated applicability to patients with stroke [15,16,17], Parkinson’s disease [18], and epilepsy [19,20], among other conditions [21,22]. EEG–fNIRS systems are of particular interest in neonatal brain research since both methods are minimally invasive, silent, and can be employed cot-side [23]. Infant studies have demonstrated that EEG–fNIRS is particularly important for understanding the development of neurovascular coupling and its (dys)regulation in association with neuropathology [24,25,26,27].

From a technical standpoint, multimodal EEG–fNIRS is commonly achieved via mechanically combining discrete, off-the-shelf EEG and fibre-based fNIRS systems [10,11,12,13,14,17,18,28,29,30]. The combination of two discrete systems poses several significant challenges: (1) the mechanical challenge of coupling the necessary EEG electrodes and fNIRS sources and detectors to the head of the subject; (2) the challenge of achieving sufficient timing precision and synchronization of simultaneous fNIRS and EEG recordings; (3) the potential for electrical crosstalk between the two systems; and (4) determining which commercial fNIRS and EEG systems can be combined. Each of these challenges is detailed in the following paragraphs.

(1) The wearability of existing discrete systems is a major challenge, as bulky optical fibres and EEG cabling and electrodes inherently compete for space on the head [14,31]. This is particularly the case for younger populations, whose small head sizes significantly limit the number of optodes and electrodes that may be used. Besides competing for space on the head, traditional fNIRS optical fibres are rarely suitable for long-term monitoring or wider applications outside of a hospital/laboratory environment. Significant progress toward single-modal wearable EEG and fNIRS technologies has been made [8,9], but progress on mechanically integrated multimodal systems is less evident.
(2) Nonintegrated EEG–fNIRS systems require an external mechanism to time-lock the acquired signals [28]. Time-locking signals consists of labelling the acquired data with marker signals or flags, and recorded signals from separate modalities are synchronized offline according to those markers. However, some time delay between signals may still be present since each instrument will independently digitize the analogue signals, each with their own specific sample rates, and based on independent clock sources with individual jitter [32].
(3) When combining electrically separate fNIRS and EEG systems, crosstalk between systems is a serious concern. Many current fNIRS system designs include laser diode (LD) or light-emitting diode (LED) sources that are integrated into optodes (or wearable modules) placed on the head. The LD/LED driving currents are often pulsed, sine-wave modulated, or square-wave modulated to permit phase sensitive demodulation of the detected signal to increase the signal-to-noise ratio (SNR) of the resulting intensity measurements [32]. Furthermore, to obtain high channel counts, fNIRS systems are typically frequency-multiplexed [33] or time-multiplexed [34], such that difference sources have different patterns of driving current. Existing methods for achieving modulation and multiplexing can create electrical crosstalk between these rapidly switching currents in fNIRS optodes and the sensitive measurements of electrical potential difference across pairs of EEG electrodes [32]. One study found that crosstalk between frequency-multiplexed fNIRS systems and EEG typically occurs outside of the EEG spectrum of interest (0.1–40 Hz) and thus can be suppressed with appropriate low-pass filters [32]. However, for time-multiplexing fNIRS systems, source switching may well occur within the EEG spectrum of interest, posing constraints on their integration.
(4) In several cases, manufacturers and/or distributors can already provide discrete fNIRS and EEG systems that can be used simultaneously. However, as every EEG and fNIRS system will have pros and cons, pre-established pairings can limit a researcher’s options. When choosing an EEG system to combine with an fNIRS system, there are multiple characteristics that must be considered. For example, there are advantages and disadvantages associated with each of the different types of EEG electrodes: dry electrodes vs. wet electrodes, and active vs. passive electrodes. Dry electrodes are placed in direct contact with the scalp, but typically have higher impedance values and are more sensitive to motion artifacts [35]. Wet electrodes (typically of Ag/AgCl metal) require application of an electrolyte gel or conductive paste to the scalp to facilitate the transduction of the ionic currents between the skin and the electrode. Wet electrodes achieve low impedance and are less sensitive to motion artifact, yet they are not feasible for long-term monitoring as the conductive gel dries over time and impedance deteriorates [36]. Active electrodes have a preamplification module immediately after the conductive material between the skin and the electrode [37]. Preamplification, i.e., use of a front-end amplifier, typically reduces noise in the EEG signal, but generally active electrodes are larger than passive electrodes, and thus require more space on the head or in a headcap. When combining EEG with fNIRS, positioning of EEG electrodes at the appropriate 10–20 points also may not be achievable depending on the positioning of fNIRS optodes.

A fully integrated, wearable EEG–fNIRS system would be the most elegant solution to overcome these shortcomings of current discrete systems [31,32]. Note, the use of term “integrated” indicates that the EEG–fNIRS system should be both mechanically and electrically integrated. Such fully integrated, or “hybrid”, systems possess a common circuit architecture and control module, thereby eliminating the potential for time delay between the signals. Integrated EEG–fNIRS systems are also designed to minimize crosstalk between the two subsystems, such as by setting the fNIRS LD/LED current switching frequency to above the EEG frequency band of interest [32]. Wearability of such a system is also essential to allow for patient/subject comfort, long-term monitoring, and real-world applications. Some form of mechanical integration of electrodes and optodes at the head is likely to be critical to achieve both wearability and the sufficient measurement densities for both EEG and fNIRS.

This review aims to describe the current status of wearable, integrated EEG–fNIRS technologies, and the advantages and limitations of each. We aim to provide direction for future projects to develop improved wearable, integrated systems. The remainder of this paper is organized as follows: Section 2 describes the strategy of our literature review and the criteria of identification and classification of published papers. Section 3 reviews the key developments towards wearable, integrated EEG–fNIRS technologies to date and describes the main characteristics of the identified systems. Section 4 evaluates several of the available types of EEG electrodes and front-end amplifiers and assesses their relative ease of integration with fNIRS. Finally, the key summary points of the review are discussed within the context of areas of future advancements.

## 2. Identified Publications

Our search strategy for articles to include in this review was as follows: Google Scholar and Web of Science search engines were used for keyword searches (near-infrared spectroscopy (NIRS) OR fNIRS) AND (EEG OR electroencephalography). Results were then manually screened to determine whether the technologies described were fully (both mechanically and electrically) integrated, wearable EEG–fNIRS technologies. Only articles that described continuous-wave (CW) fNIRS systems were included, as there are currently no other modalities of integrated EEG–fNIRS technologies well demonstrated in peer-reviewed publications. This search strategy resulted in 10 key publications, all of which are described in the sections below.

While the technologies described in these works vary significantly, we have attempted to classify these systems into two broad categories on the basis of their manufacturing processes. The technologies presented in [38,39,40,41,42,43] were developed purely using commercially available discrete components, and these off-the-shelf discrete components were assembled together on printed circuit boards (PCBs) to complete the integrated system. Thus, we categorize these technologies as “discrete components-based technologies”, details of which are summarized in Table 1. In contrast, the key components/functional blocks that were employed in the integrated systems in [44,45,46,47] were fabricated in a custom-designed microchip using complementary metal–oxide–semiconductor (CMOS) microchip processes. Using this approach, the majority of the components and functional blocks of the system are implemented in a standalone microchip with an ultra-small footprint. Throughout this paper, these technologies are simply grouped as “microchip-based technologies”.

## 3. State-of-the-Art, Wearable, Integrated EEG–fNIRS Devices

### 3.1. Discrete Components-Based Technologies

In 2013, Safaie et al. proposed an integrated EEG–fNIRS system [38]. This system consisted of four primary parts: front-end EEG recording electronics, head-mounted optoelectronics, a control unit, and a laptop. In the fNIRS block, there were eight dual-wavelength LEDs (760 nm and 850 nm, L760/850, Epitex) as sources, and four silicon diodes (ODA-6WB-500M, Optodiode) employed as detectors. This arrangement could produce up to 32 channels with a theoretical source-detector separation (SDS) in the range of 20–63 mm. A 16-bit analogue-to-digital converter (ADC) (ADS1178, Texas Instruments) was utilised for fNIRS data acquisition, and the overall sampling rate was 8 Hz. The EEG block supported 12 standard EEG Ag/AgCl sintered cup electrodes. A 16-channel biopotential front-end amplifier was chosen to acquire EEG signals which could measure electrode–skin impedance in the range of 0–100 kΩ. A 16-bit ADC (AD7985, Analog Devices) was multiplexed and utilised to acquire the recorded EEG signals. The sampling rate of EEG signals was 1024 Hz. A custom-designed optoelectronic “patch” was implemented to integrate both EEG and fNIRS components together, with dimensions of 35 × 80 × 10 mm^3^ and weight of 90 g. The control unit and laptop were utilised for data acquisition and transmission. This system can be wirelessly operated via an in-built Bluetooth module, demonstrating a total system power consumption of ~400 mW.

This system successfully integrated EEG and fNIRS modules into a wearable system. It achieved wireless operation and a relatively lightweight form factor. However, there are also several concerns with this work. First, the fNIRS module claimed a promising SDS (up to 63 mm) and a theoretical dynamic range (up to 198 dB), yet no demonstrations were conducted at longer separations to validate the data quality and practical dynamic range. Besides, the capacity for (3D) imaging of the fNIRS module was unclear. Moreover, the EEG demonstrated a relatively limited measuring range of electrode–skin impedance, which is inadequate for use of dry electrodes. Besides this, the EEG module and fNIRS module separately employed two different ADCs for data acquisition. This design not only additionally increased the size and power of the system, but also could cause concern for timing precision (between markers in the fNIRS and EEG data streams). More critically, for both fNIRS and EEG modules, this system applied extensive cabling for analogue signal transmission from EEG electrodes and optical detectors to the distant control module, which could be inconvenient for practical applications as well as make the system vulnerable to radio frequency (RF) noise. In addition, the scalability of current ergonomic design of the system is limited.

**Figure 1:**
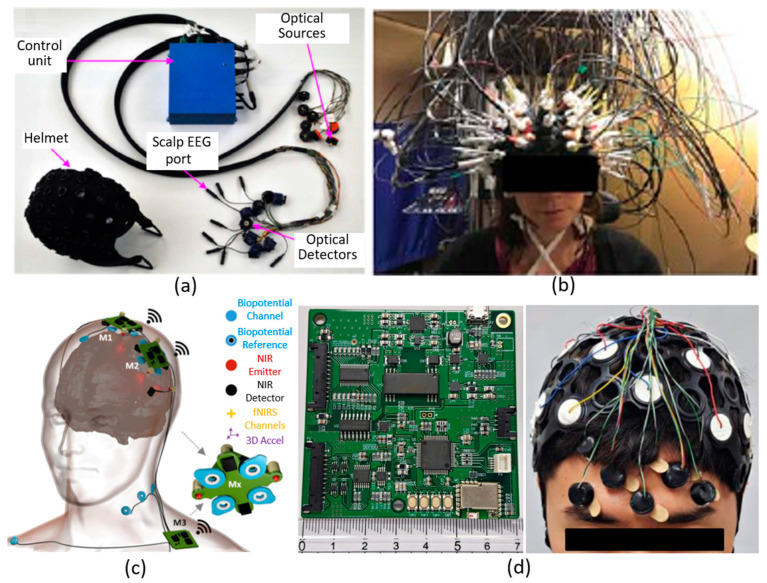
Examples of discrete components-based, integrated EEG–fNIRS systems. (**a**) The wireless, integrated EEG–fNIRS system described by Sawan et al. It consists of a helmet to house the front-end optical and electrical components, a distant control unit for system control and data transmission, and cabling. This figure was taken and modified with permission from [39]. (**b**) The system with 128 fNIRS channels (32 sources, 32 detectors) and 32 EEG channels described by Kassab et al. Extensive cabling was utilised in this multichannel system. This figure was taken with permission from [42]. (**c**) The modular, integrated EEG–fNIRS system described by von Luhmann et al. It is comprised of three individual modules, producing 13 fNIRS and 8 EEG channels. This figure was taken with permission from [40]. (**d**) The dry electrode-based, integrated EEG–fNIRS system described by Lee et al., consisting of a cap to position eight optodes (2 sources and 6 detectors), two custom-designed control boards, and cabling. This figure was taken with permission from [43].

The same year, building upon their prior work [41], Sawan et al. developed a wireless, wearable EEG–fNIRS integrated system [39], as shown in Figure 1a below. This system contained two primary parts: a wearable helmet and a control unit. In the helmet, there were eight sources, eight detectors, and eight EEG recording sites. Dual-wavelength LEDs (735 nm and 850 nm, Epitex) were chosen as sources, and avalanche photodiodes (APDs) (S2384, Hmamatsu) were selected as detectors. Particularly, a high bias voltage (150 V) was applied to the APDs so as to achieve satisfactory sensitivity. Theoretically, each light source could be coupled with four surrounding detectors, thus this system could produce up to 32 fNIRS channels, but the actual value of SDS was not noted. The front-end LED driving circuits and detection circuits were both fitted into small-size circular PCBs (130 mm^2^). However, the physical implementation of the EEG electrodes and their front-end electronics were unclear. In the control unit, three different stack layers of PCBs were implemented to realize system control, power, and data transmission. A 16-bit ADC was utilized to acquire both fNIRS and EEG data, with sampling rates of 20 Hz and 320 Hz, respectively. A Bluetooth module was embedded into the control unit to achieve wireless data transmission. The control unit contained a Li-ion battery (10Ah-7.4 V, 76 × 80 × 40 mm^2^, 400 g) to act as supply power for the overall system, with a measured power consumption of 2.2 W. The overall dimensions of the control unit were 160 × 130 × 82 mm^2^, and the weight was 800 g. This system has recently been further upgraded to include more sources, detectors, and electrodes, achieving 128 fNIRS channels (32 sources, 32 detectors) and 32 EEG channels, but with increased size and weight, along with a more extensive cabling [42], as shown in Figure 1b.

This work demonstrated a wireless, wearable, integrated EEG–fNIRS system. A shared ADC architecture was implemented to simultaneously acquire fNIRS and EEG data, and this setting could potentially improve the timing precision of multimodal data acquisition. However, similar to Safaie et al.’s work presented above, this system employed highly cumbersome cable connections between front-end components and the control unit. This setting would limit its practical and longer-term applications, and, more critically, could incur external RF noise during the analogue data transmission between the helmet and the control unit, and then consequently bias the data quality. This work explained the implementation of head-mounted optoelectronics, but the realization of EEG electrodes and associated front-end electronics was not clearly demonstrated. Moreover, the measuring range of electrode–skin impedance was not presented either, so no clear information about the compatibility with different types of electrodes (i.e., wet, dry, or both) could be provided for users/readers. Besides this, the SDS value of fNIRS data was also unclear, and the possible imaging capability was also not demonstrated. In addition, the crosstalk effect between optics and electronics of this system was not evaluated.

In 2017, von Luhmann et al. developed a modular system architecture to achieve an integrated multichannel EEG–fNIRS system [40], as shown in Figure 1c above. Each module was equipped with two dual-wavelength LEDs (750 nm and 850 nm, L750/850-04A, Epitex) as NIR light sources, and employed two silicon photodiodes (OPT101, Burr-Brown) as NIR detectors. The SDS was arranged around 35 mm. Each module could produce up to six fNIRS channels (four inter-module channels, and two theoretical cross-module channels). A 24-bit ADC (ADS 1299, Texas Instruments) was utilised for fNIRS data acquisition. The sampling rate of the fNIRS data was set as 16.6 Hz. The noise equivalent power (NEP) of the detection circuitry was characterised, obtaining minimum value of 4.77 pW at 850 nm and maximum value of 5.92 pW at 750 nm. In each module, up to six theoretical EEG channels could be generated. The 24-bit ADC used for fNIRS data acquisition was also utilised for EEG data acquisition simultaneously, achieving a sampling rate of 500 Hz. Due to the high input impedance (1 TΩ) and common-mode rejection ratio (–110 dB), both wet and dry EEG electrodes could be (theoretically) suitable for use. A three-axis accelerometer (ADXL343, Analog Devices) was also embedded into each module for local movement monitoring. Besides, a Bluetooth module was implemented for wireless data transmission. The module can be powered by a small-size Li-ion battery (300 mAh, 28 × 34 × 0.6 cm^3^), and the power consumption of each module was around 360 mW. This modular architecture can be theoretically scaled up to four modules, and in this presented work, a three-module fNIRS+EEG system was demonstrated (as shown in Figure 1c), producing 13 fNIRS and 8 EEG channels.

This work proposed a modular, wearable, multimodal system that can achieve fNIRS measurement, EEG recording, and motion monitoring concurrently in a single module. Besides this, they created a shared ADC architecture which could potentially improve time precision and data synchronisation. In addition, battery powering and wireless operation were also implemented for each module. This system demonstrated several merits, however, there are still some key limitations. First, the capability for (3D) brain imaging was yet demonstrated. Particularly, (most of) the SDS were fixed at 35 mm, which indicates that only sparse spatial sampling could be produced. Moreover, the footprint of each module was large (42 × 42 mm^2^), which constrained the wearability and comfort of the system. Furthermore, the scalability of this modular design was limited, and it is challenging to use this system for whole-scalp sampling. In addition, the weight of the module was unclear.

In 2019, Lee et al. proposed a dry electrode-based integrated EEG–fNIRS system [43]. A block diagram of the system is shown in Figure 1d. In this system, two dual-wavelength LEDs (730 nm and 850 nm, OE-MV7385-P, Opto ENG) were utilised as fNIRS sources, and six silicon photodiodes (OPT101, Texas Instruments) were employed as fNIRS detectors. This arrangement generated eight fNIRS channels in the area of 9 cm × 3 cm, with a fixed SDS of 27 mm. An MOSFET-based LED driver was implemented to modulate LED illuminations with fine-tuned intensity. A 16-bit ADC (ADS8688A, Texas Instruments) was embedded to acquire the eight-channel fNIRS data with a sampling rate of 5 Hz. A custom-designed prototype of dry electrodes was implemented for EEG recording. Each dry electrode consisted of 18 spring-loaded probes (SK100R, Leeno Industrial Inc.) so as to provide contact with the subject’s scalp. Sixteen dry electrodes were employed in this system for EEG recording. Two 24-bit ADCs (ADS1299, Texas Instruments) were dedicated to being used for 16-channel EEG data recording, with a sampling rate of 250 Hz. Two four-layer PCB-based control units (70 mm × 70 mm each) were fabricated for data acquisition for all the EEG and fNIRS channels and overall system control. This system can be battery powered, and the average power consumption of each recording channel was about 18.8 mW.

Although this work successfully integrated fNIRS and EEG modalities into a standalone system, this system has several limitations similar to the systems of [48,49,50] (Table 2). First, it utilised extensive cabling between optodes/electrodes and the distant control unit, which was bulky and cumbersome, and limited its subject comfort and long-term wearability. More critically, this could potentially make both fNIRS and EEG signals vulnerable to environmental RF noise. Moreover, this system demonstrated a fixed SDS of only 27 mm, thus, only sparse sampling can be achieved using this device. In addition, the total weight of the system was unclear. Overall, this newly developed system did not demonstrate obvious advancements over previous designs in [38,39,40,41,42].

Of note, there are several commercially available wearable fNIRS systems [48,49,50] that claimed that they can be integrated with EEG, yet these systems only mechanically integrated fNRIS optodes with EEG ports on the cap. These systems still employed separated electronic designs, far from a fully integrated system.

### 3.2. Microchip-Based Technologies

The section above described the integrated EEG–fNIRS technologies, which fully consisted of off-the-shelf discrete components. An alternative approach is to implement and integrate the primary fNIRS measurement circuitry and EEG recording circuitry into a single microchip using custom-designed CMOS integrated circuits (IC). This strategy can potentially allow for a smaller footprint, ultralight weight scale, lower noise and power, better signal quality, and improved timing precision.

**Figure 2:**
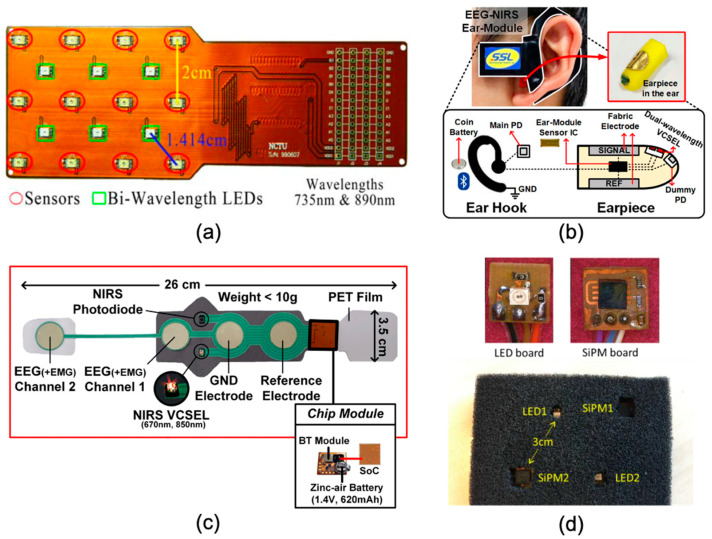
Examples of microchip-based technologies. (**a**) The CMOS-based integrated EEG–fNIRS system developed by Chua et al. This system uses a bendable PCB to house 6 dual-wavelength LEDs and 12 detectors. This figure was taken with permission from [44]. (**b**) The SoC-based EEG–fNIRS ear-module system. The SoC, source, and electrode are embedded in an earpiece while the main photodiode, battery, and Bluetooth module are located at an ear hook. This figure was taken with permission from [45]. (**c**) The integrated EEG–fNIRS system for anaesthesia depth monitoring developed by Ha et al. The SoC with Bluetooth module and battery are embedded on a flexible PCB section and a polyethylene terephthalate (PET) film is fabricated to house the sources, detectors, EEG electrodes, and accessory components. This figure was taken and modified with permission from [46]. (**d**) The CMOS-based integrated multimodal EEG–fNIRS–EIT systems developed by Xu et al. A dual-wavelength LED is employed as an optical source, and a SiPM is utilised as an optical detector. This figure was taken with permission from [47].

In 2011, Chua et al. proposed a CMOS-based, highly integrated EEG–fNIRS system [44]. In this system, six dual-wavelength LEDs (735 nm and 890 nm) were equipped as fNIRS sources, and twelve detectors were positioned adjacent to these LEDs, as shown in Figure 2a below. The fNIRS sensor array was implemented on a bendable PCB. The SDS was set as 14.14 mm, and this setting generated 24 source-detector channels. A 10-bit ADC was implemented to achieve a 1 Hz sampling rate for fNIRS data. Another 10-bit ADC was employed to record EEG data at a sampling rate of 128 Hz. However, the number of EEG channels and electrodes were not depicted. A UMC 65 nm CMOS technology was used to fabricate the microchip that included an fNIRS data processor, a four-channel independent component analysis (ICA) (for EEG recording), and a heart rate variability (HRV) analysis processor. The dimensions of the die were 1317 um × 1317 um, and the active area (core size) was 680 um × 680 um. The simulated power consumption of the microchip was 3.6 mW.

This work presented a microchip-based multimodal platform for simultaneous fNIRS and EEG measurement and data acquisition. Particularly, it integrated an fNIRS data processor, an ICA engine, and a HRV processor into a compact chip layout. However, there are several concerns with this work. First, this work did not clearly demonstrate how the fabricated chip can be employed as a practical multimodal system. Secondly, the implementation of EEG front-end and electrodes was unclear, and the number of EEG channels was also not provided. Moreover, the possible crosstalk between fNIRS signals and EEG signals was not taken into consideration. Furthermore, this work demonstrated a bendable PCB-based sensor array, but it was unclear how this array can be physically and practically applied onto patients/subjects. Besides this, the SDS of the array was fixed at 14.14 mm, and this indicates that it would be challenging to apply this system to produce (3D) images. In addition, all the data presented in this work were based on simulation, while measurement validations of the system performance were not demonstrated.

In 2016, Ha et al. proposed an integrated EEG–fNIRS ear-module system [45], as shown in Figure 2b. A system-on-chip (SoC) architecture was implemented, containing three primary blocks: (1) a sophisticated front-end transimpedance amplifier, (2) a tuneable VCSEL driver, and 3) a reconfigurable impedance boosting loop for EEG recording. The SoC was then integrated into a wearable ear-module device, which primarily included an ear hook and an earpiece. In this ear-module device, there was one dual-wavelength VCSEL (670 nm and 850 nm) utilised as optical source and two photodiodes employed as detectors. In particular, one of the photodiodes was closely placed adjacent to the VCSEL (located at an optode board) to act as a dummy detector while another photodiode located at the ear hook served as the main detector. An 11-bit ADC was realized and shared to record both fNIRS and EEG data. A dedicated VCSEL driver was implemented with optimized SNR, and a 7-bit digital-to-analogue converter (DAC) was incorporated with the VCSEL to modulate the light intensity. For EEG recording, a reconfigurable impedance boosting loop was proposed that can potentially achieve input impedance (of EEG) up to 5.4 GΩ. This ear-module SoC was fabricated in a 110 nm 1-poly 6-metal CMOS process. The die size area was 450 um × 2250 um. The maximum power consumption of this ear-module SoC was 46.2 mW.

Although this system demonstrated some interesting features, such as the SNR-optimizing tuneable VCSEL driver for NIR light illumination and the reconfigurable impedance boosting loop for ultra-high EEG input impedance, there are some concerns with this work. First, it was unclear whether this ear-module design can be scaled up to multiple modules for brain monitoring. Secondly, some key parameters of fNIRS were not provided, such as the SDS and the dynamic range, thus it is challenging to evaluate the performance of fNIRS functionalities. Besides this, the sampling rates of fNIRS and EEG measurements were not noted. Moreover, the power consumption of this ear module was relatively high, and the total power consumption could be a concern if the system was scaled up to include more modules. In addition, the weight of this system was unclear.

One year later, the same group in [45] above proposed a different integrated EEG–fNIRS system, using more advanced 65 nm CMOS technology [46]. Figure 2c shows the EEG–fNIRS integrated system for anaesthesia depth monitoring. In the proposed monitoring system, a dual-wavelength VCSEL (670 nm and 850 nm) was utilised as a source, and a photodiode was employed as an NIR detector. There were four electrodes (two recording electrodes, one ground electrode, and one reference electrode) to obtain two EEG channels. A polyethylene terephthalate (PET) film and a flexible PCB board were fabricated to house the fabricated SoC, NIRS sources and detectors, EEG electrodes, and accessory components, achieving a lightweight form factor (<26 g) and a relatively small profile (35 mm × 260 mm) of this monitoring system. The core SoC, containing the main circuitry for EEG–fNIRS recording, data acquisition, and system control, was embedded on the flexible PCB section of the wearable monitoring system, as shown in Figure 2c. The proposed SoC contained five primary functional blocks: (1) EEG recording module; (2) NIR detection module; (3) VCSEL driving circuitry; (4) ADC; and (5) digital control/communication module. In the EEG module, a reconfigurable impedance boosting loop was adapted from [45] to achieve a 1 GΩ input impedance. A logarithmic transimpedance amplifier (TIA) was implemented in the NIR detection module so as to obtain a dynamic range of 60 dB. A 6-bit DAC was employed in the VCSEL driving circuitry to modulate the drive current from 0 to 17.2 mA to modulate the light intensity. A shared 12-bit ADC was utilised to simultaneously fetch both fNIRS and EEG data, with a sampling rate of 20–80 Hz and 2 kHz, respectively. A digital module was realized in the SoC to achieve communication, buffering, filtering, and system control. This SoC was fabricated in a 65 nm CMOS process, with a compact layout of 4000 um × 4000 um. A Bluetooth module was embedded in the system to support wireless operation. The maximum power consumption of the system was 25.2 mW.

This work demonstrated a sophisticated SoC for integrated EEG–fNIRS monitoring. It realized a lightweight, wearable system for accurate anaesthesia depth monitoring. Though promising, this work has several limitations similar to the work in [45] above, mainly including (1) uncertainty of the scalability for large-area, or even whole scalp, monitoring; (2) unclear information for SDS; (3) relatively restricted dynamic range (60 dB) for NIR light detection that can limit wider applications of this system.

In 2018, Xu et al. proposed a CMOS-based EEG–fNIRS–electrical impedance tomography (EIT) integrated system [47]. fNIRS, EEG, and EIT functional blocks were implemented and integrated into a microchip. In the fNIRS functional block, a dual-wavelength LED (735 nm and 850 nm) was utilised as an NIR source, and a silicon photomultiplier (SiPM) (with 4871 square microcells, 5.1 mm × 5.1 mm) was employed as an NIR detector, with a bias voltage of 30 V and a power consumption of ~1.5 mW. A 12-bit ADC was utilised to fetch the fNIRS data. Figure 2d shows an fNIRS patch with sources and detectors. In this fNIRS patch, there were two source locations and two detector locations, with a fixed SDS of 30 mm. For EEG recording, it achieved an input impedance of 720 MΩ, but no practical implementation of EEG electrodes was demonstrated in this work. The presented microchip was fabricated using a 180 nm CMOS technology, with a compact chip layout, a small die size (4000 um × 4000 um), and a low power consumption of 665 uW (chip only). 

This presented work achieved an integrated design for multimodal EEG–fNIRS–EIT monitoring and demonstrated an advanced microchip design. Despite its merits, there are still some concerns with this work. Firstly, this microchip design can only support one fNIRS channel and one EEG channel; more chips would be needed with more measurement channels. From the primary fNIRS patch design shown in Figure 2d, the scalability of this design is unclear, as well as how one could extend this microchip to a practical, modular, wearable EEG–fNIRS system. Besides this, the fixed SDS setting could limit the sampling capability of the system. Long-term wearability and subject comfort could be additional concerns. Moreover, although the utilisation of SiPM could potentially improve the NIR detection sensitivity, the required relatively high bias voltage (of 30 V) could be a concern when mounting the optodes on subjects’ heads. In addition, the possible crosstalk between fNIRS and EEG was not properly taken into account.

## 4. EEG Electrodes and Front-End Amplifiers

The focus of this section is mainly to summarize the types of EEG electrodes and associated front-end amplifiers utilised in current wearable integrated EEG–fNIRS systems. To note, there are relevant systematic reviews on EEG background [51,52], wearable EEG [9], electrode type [53,54], electrode materials [55], and EEG signal processing [56]. This section does not aim to replicate these reviews. Instead, it aims to complement them by providing an overview of the use of electrodes and amplifiers in integrated EEG–fNIRS technologies.

**Figure 3:**
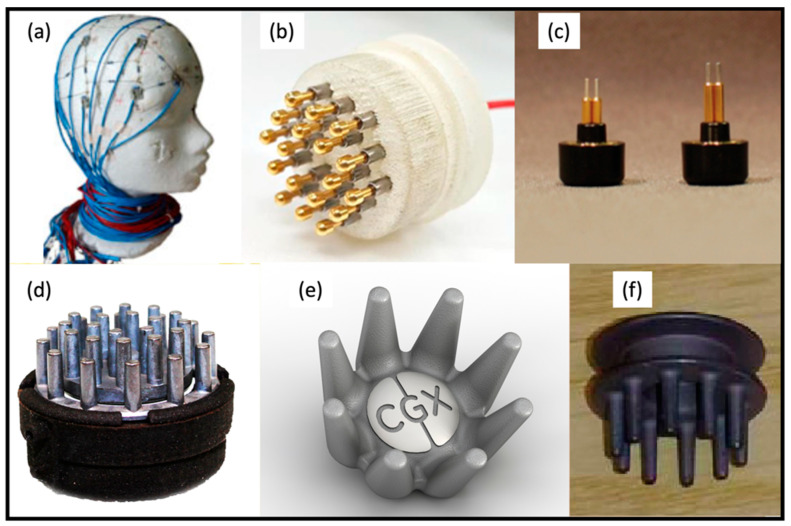
(**a**) The wet AgCl ring electrodes used in the system developed by von Luhmann et al. This figure was taken with permission from [40]. (**b**) The custom-designed prototype of spring-loaded dry electrodes developed by Lee at al. Each electrode contains 18 spring-loaded probes. This figure was taken with permission from [43]. (**c**) A commercial dry electrode used in a single-modal EEG, described by Kam et al. This figure was taken with permission from [59]. (**d**–**f**) Examples of commercial dry fingered EEG electrodes: (**d**) wearable sensing [64], (**e**) CGX [65], (**f**) neuroelectrics [66]. These figures were taken with permission from [64], [65], and [66], respectively.

In general, EEG electrodes can be classified into whether they are wet or dry, and whether they are active or passive. This leads to four possible combinations: passive wet, active wet, passive dry, and active dry [57,58]. Table 3 summarizes the types of EEG electrodes utilised in current wearable integrated EEG–fNIRS systems, along with the information about electrode materials, sizes of electrodes, and the number of electrodes applied in the system. Figure 3a,b below also show the actual patterns of electrodes used in [38] and [43], respectively. It can be seen from Table 3 that most of the systems can (theoretically) support dry electrodes. Though promising, limited information on electrode materials, size, and number of electrodes applied in the system were presented, which makes it difficult to thoroughly evaluate their performance.

Apart from these electrodes summarized in Table 3, there have also been various types of EEG electrodes commercially available and that have been applied in single-modal EEG systems. For example, Kam et al. [59] demonstrated a type of passive dry electrodes (Nielsen, NY, USA) with two different lengths of metal pins (Figure 3c) that can potentially accommodate with different hair types. In recent years, dry fingered electrodes have become prevalent, particularly for applying electrodes in haired regions [9]. Figure 3d–f show some examples of current dry fingered EEG electrodes utilised in commercially available systems [9,60]. Typically, these electrodes use the finger structures to push apart the hair so as to make a contact with the scalp. Despite the success, the conformability (to the curved human scalp) and subject comfort of these electrodes still can be improved. Besides this, how to fit these relatively bulky electrodes into a miniaturized, compact wearable fNIRS module/functional blocks [8,61,62,63] remains unclear.

Table 3 also summarizes the key parameters of front-end amplifiers used for EEG recording in [38,39,40,43,44,45,46,47]. Most of these amplifiers demonstrated a high input impedance that is compatible with dry electrodes. Considering the input noise, most of the IC-based amplifiers achieved low input noise (<=1.2 uVrms), while there is still some room for improvement with the discrete amplifiers used in [38,39,40,43]. Note, although the ADS1299 (Texas instruments, USA) used in [40,43] (containing front-end amplifier and multichannel ADC) was a discrete component, it still demonstrated overall fine performance, such as high input impedance, multichannel data acquisition, and satisfactory timing precision, which could facilitate simultaneous multichannel data acquisition of fNIRS and EEG. In recent years, some works on IC-based EEG amplifiers have demonstrated encouraging performance [67,68,69]. However, these amplifiers have not yet been implemented and verified in any integrated EEG–fNIRS system.

## 5. Discussion

Multimodal EEG–fNIRS is highly advantageous in that it allows for the concurrent assessment of electric and hemodynamic brain activity. EEG–fNIRS has been increasingly applied in multiple sectors, such as BCI [10,11,12,13,14], clinical neurology [15,16,17,18,19,20,21,22], and neurorehabilitation [70,71]. In the evolution of this field, the development of wearable, integrated EEG–fNIRS technology is a critical next step. 

Our review presents the state-of-art in wearable, fully integrated EEG–fNIRS systems. To date, there have been several integrated systems developed using off-the-shelf discrete components. Despite the success, these technologies are subject to the following limitations: (1) large size, heavy weight, and limited wearability and subject comfort; (2) crosstalk between optical and electrical signals; (3) inaccurate timing precision/synchronization for simultaneous fNIRS and EEG recording; (4) low signal quality and/or limited capability of long-term recording of EEG electrodes.

To overcome some of these limitations, an emerging trend has been the use of (mechanically and electrically) integrated designs via microchips. Microchip technologies hold several key advantages, including a small footprint, low power consumption, better timing synchronization between fNIRS and EEG measurements, and potentially improved signal quality with reduced noise and crosstalk [32,72]. Several attempts at a microchip-based, wearable, integrated EEG–fNIRS system have been made in the last ten years [44,45,46,47]. In addition to IC designs, other emerging trends include modular, lightweight, and flexible designs, wireless capabilities, shared ADCs for timing precision between optical and electrical data, and consideration of both wet and dry EEG electrodes.

Looking towards the future of EEG–fNIRS systems, investigations into ergonomic designs (i.e., wearable, miniaturized, lightweight, flexible) will continue to remain of key interest for long-term monitoring, wearability, and subject comfort. Improvements in resolution, high-speed ADCs, sensitive amplifiers, and precise logic control will be necessary to obtain the large dynamic range needed for high channel count fNIRS systems [8,62,63,64]. Consequently, more advanced data interfaces and control schemes would be required to ensure a large channel count, large dynamic range, and high-speed data acquisition and transmission. Additionally, dry EEG electrodes with high gain and high input impedance would probably be a preferred choice for long-term, regular monitoring. Dry electrodes may be increasingly incorporated into designs as they are lightweight, small, flexible, and have the capacity to make stable contact with the scalp, including through hair. To achieve these goals, extensive efforts are needed in electronic design, mechanical analysis, software development, and precise fabrication with biocompatible materials. 

## 6. Conclusions

For the multimodal assessment of brain function, current EEG–fNIRS systems possess several limitations, but their future development has the potential to revolutionize how we study and care for the brain. This review provides an in-depth analysis of the current state of wearable, integrated EEG–fNIRS systems, describing their features, advantages, and disadvantages. Now, the picture of the “ideal” new-generation, wearable, integrated EEG–fNIRS technology becomes clearer. This system should be designed with an ultra-low profile, a lightweight form factor, a high channel count, a large dynamic range, good subject comfort, robust contact with the (haired) scalp, precise timing synchronization, minimal crosstalk, stable data transmission, and long battery life. With multidisciplinary efforts from engineers, medical physicists, and clinicians, this “ideal” technology could become possible in the next few years, and it would potentially have wide-reaching implications for sectors including neuroscience, psychology, clinical neurology, BCI, neurorehabilitation, and personalized healthcare.

## Figures and Tables

**Table 1:** Characteristics of key wearable, integrated EEG–fNIRS technologies. Note: the use of “n/a” indicates that the specific characteristic (e.g., chip area) is not applicable to that system. The em dash (—) symbol indicates that the information is not available.

Author, Year	Fabrication Process	Size of Head Element or Module	Size of Control Unit	Chip Area (um^2^)	Weight (g)	No. of S/D	No. of Opto-Channels	Wave- Length (nm)	SDS (mm)	No. of EEG Channels	EEG Electrode Position on Scalp	NIRS/EEG Recording Resolution (bit/bit)	ADC Setting	fNIRS/ EEG Sampling Rate (Hz/Hz)	Wireless Function	Power (mW)
Safaie et al., 2013 [38]	Discrete	35 × 80 × 10 mm^3^	—	n/a	90	8/4	32	760, 850	20 to 63	8	10–10 standard	16/16	Separated	8/1024	Yes	400
Sawan et al., 2013 [39]	Discrete	130 mm^2^	160 × 130 × 82 mm^3^	n/a	800	8/8	32	735, 850	31	8	—	16/16	Shared	20/320	Yes	2200
Kassab et al., 2018 [42]	Discrete	95 mm^2^	120 × 90 × 70 mm^3^	n/a	850	32/32	128	735, 850	~30	32	—	16/16	Separated	20/320	Yes	2600
von Luhmann et al., 2017 [40]	Discrete	42 × 42 mm^2^	—	n/a	—	6/6	13	750, 850	35	8	Dependent on where module places	24/24	Shared	16.6/500	Yes	360
Lee et al., 2019 [43]	Discrete	—	70 × 70 mm^2^ × 2	n/a	—	2/6	8	730, 850	27	16	16 locations frontal and parietal cortex as per 10–20 standard	16/24	Separated	5/250	Yes	18.8 (per chan.)
Chua et al., 2011 [44]	CMOS 65 nm	—	—	1317 × 1317	—	6/ 12	24	735, 890	14.14	—	—	10/10	Separated	1/128	No	3.6 (chip only)
Ha et al., 2016 [45]	CMOS 110 nm	—	—	450 × 2250	—	1/2	1	670, 850	—	1	Forehead (AF7) and temple (FT9)	11/11	Shared	—	No	46.2
Ha et al., 2017 [46]	CMOS 65 nm	35 × 260 mm^2^	—	4000 × 4000	<26	1/1	1	670, 850	—	2	Forehead (AF7) and temple (FT9)	12/12	Shared	20-80/2000	Yes	25.2
Xu et al., 2018 [47]	CMOS 180 nm	—	—	4000 × 4000	—	2/2	4	735, 850	30	1	—	12/15	Separated	2-512/-	No	0.665 (chip only)

**Table 2:** Summary of the advantages and disadvantages of the integrated EEG–fNIRS systems described in this review.

Author, Year	Advantages	Disadvantages
Safaie et al., 2013 [38]	32 channels fNIRS, 16 channels EEG 20–63 mm theoretical SDS Wirelessly operated via Bluetooth module Battery operated Relatively lightweight	No demonstration of data quality and practical dynamic range Capacity for 3D imaging unclear Separate ADCs for data acquisition (increases size, power, and potentially a time offset between EEG and fNIRS acquisitions) Extensive analogue cabling for EEG and fNIRS into control module Limited ergonomic scalability
Sawan et al., 2013 [39]	32 fNIRS channels, 8 EEG channels Miniaturized front-end electronics Sensitive detectors (APDs) Wireless, and battery operated Shared ADC architecture	Extensive analogue cabling for EEG and fNIRS into control module Physical implementation of EEG electrodes and compatibility of different types of electrodes unclear Imaging capability and SDS of fNIRS channels unclear High bias voltage of APD (150 V), may cause safety concerns
Kassab et al., 2018 [42]	128 fNIRS channels, 32 EEG channels Same advantages as above system	Same limitations as above system Increased size, weight, and cabling as compared to above system
von Luhmann et al., 2017 [40]	Modular design Both wet and dry EEG electrodes are theoretically suitable 3-axis accelerometer for local movement monitoring Shared ADC architecture	Limited channel numbers, only 13 fNIRS channels and 8 EEG channels Relatively limited scalability, can only be theoretically scaled up to four modules Capability of imaging not demonstrated Fixed SDS at 35 mm (sparse spatial sampling) Modules possess a large footprint Not capable of whole scalp sampling Weight of each module unclear
Lee et al., 2019 [43]	16 EEG channels Custom-designed dry EEG electrodes Battery operated	Limited fNIRS channel numbers, only 8 fNIRS channels Extensive analogue cabling for EEG and fNIRS into control module Fixed SDS of 27 mm (sparse spatial sampling) Separate ADCs Total weight of system unclear
Chua et al., 2011 [44]	Microchip-based and highly integrated 24 fNIRS channels	SDS fixed at 14.14 mm (sparse spatial sampling) Number of EEG channels not described Possible crosstalk between EEG and fNIRS channels not considered Not demonstrated on human subjects Measurement validations of the system not demonstrated
Ha et al., 2016 [45]	System-on-chip (SoC) architecture Wearable ear-module design Shared ADC architecture SNR-optimizing tuneable VCSEL driver for NIR light illumination Reconfigurable impedance boosting loop for ultra-high EEG input impedance	Limited channel numbers, only 1 fNIRS channel and 1 EEG channel in each module The scalability of the ear-module design is unclear SDS and the dynamic range of fNIRS channels not provided Sampling rates of fNIRS and EEG measurements not noted Relatively high power consumption Weight of system not noted
Ha et al., 2017 [46]	Microchip-based design Lightweight, small profile Wireless operated Shared ADC architecture Reconfigurable impedance boosting loop for ultra-high EEG input impedance Demonstrated in human patients for anaesthesia deep monitoring	Limited channel numbers, only 1 fNIRS channel and 2 EEG channels in the system Scalability for large area monitoring not clear SDS of fNIRS channels unclear Relatively restricted dynamic range (60 dB) for NIR light detection
Xu et al., 2018 [47]	Microchip-based design Integrated system included electrical impedance tomography Sensitive detectors (SiPMs) High input impedance for EEG Compact layout Low power consumption	Only 4 fNIRS channels and 1 EEG channel Scalability of design to wearable system unclear Fixed SDS of 30 mm Implementation of EEG electrodes not demonstrated Not demonstrated on human subjects High voltage bias of SiPM (30 V) could be of concern when mounting on subjects’ heads Possible crosstalk between EEG and fNIRS systems not considered

**Table 3:** Characteristics of EEG electrodes and front-end amplifiers used in key wearable, integrated EEG–fNIRS technologies. Note: the use of the em dash (—) symbol indicates that the information is not available.

Author, Year	Electrode Type	Electrode Materials	Size of Electrode	Input Noise (*u*V_rms_)	Input Impedance	CMRR
Safaie et al., 2013 [38]	Active Wet	Ag/AgCl	Diameter: 8 mm	2	100 KΩ	—
Sawan et al., 2013 [39]	—	—	—	—	—	—
Kassab et al., 2018 [42]	—	—	—	—	—	—
von Luhmann et al., 2017 [40]	Active Wet (Practical) / Active Wet and Dry (Theoretical)	AgCl	—	<0.28	1 TΩ (Theoretical)	110 dB
Lee et al., 2019 [43]	Active Dry	—	—	0.141	1 TΩ (Theoretical)	110 dB
Chua et al., 2011 [44]	—	—	—	—	—	—
Ha et al., 2016 [45]	Active Dry	Fabric	—	0.65 @ 0.5–100 Hz	5.4 GΩ	—
Ha et al., 2017 [46]	Active Dry	—	—	0.48 @ 0.5–100 Hz	1 GΩ	>110 dB
Xu et al., 2018 [47]	Active Dry (Theoretical)	—	—	1.2 @ 0.5–100 Hz	720 MΩ	100 dB

## Data Availability

Not applicable.
